# Controlled ovarian stimulation for oocyte preservation in childhood cancer survivors who have undergone chemotherapy

**DOI:** 10.1093/hropen/hoaf023

**Published:** 2025-05-09

**Authors:** Moran Shapira, Dror Meirow, Dani Raved, Leyla Levy, Noah Gruber, Dalit Modan-Moses, Raoul Orvieto, Myriam Safrai

**Affiliations:** Obstetrics & Gynecology Department, Fertility Preservation Center, Sheba Medical Center, Ramat Gan, Israel; Obstetrics & Gynecology Department, IVF Institute, Sheba Medical Center, Ramat Gan, Israel; Faculty of Medical and Health Science, Tel Aviv University, Tel Aviv, Israel; Obstetrics & Gynecology Department, Fertility Preservation Center, Sheba Medical Center, Ramat Gan, Israel; Obstetrics & Gynecology Department, IVF Institute, Sheba Medical Center, Ramat Gan, Israel; Faculty of Medical and Health Science, Tel Aviv University, Tel Aviv, Israel; Faculty of Medical and Health Science, Tel Aviv University, Tel Aviv, Israel; Department of Pediatric Hematology–Oncology, Safra Children’s Hospital, Sheba Medical Center, Ramat Gan, Israel; Faculty of Medical and Health Science, Tel Aviv University, Tel Aviv, Israel; Faculty of Medical and Health Science, Tel Aviv University, Tel Aviv, Israel; Pediatric Endocrinology and Diabetes Unit, The Edmond and Lily Safra Children’s Hospital, Sheba Medical Center, Tel-Hashomer, Israel; Faculty of Medical and Health Science, Tel Aviv University, Tel Aviv, Israel; Pediatric Endocrinology and Diabetes Unit, The Edmond and Lily Safra Children’s Hospital, Sheba Medical Center, Tel-Hashomer, Israel; Obstetrics & Gynecology Department, Fertility Preservation Center, Sheba Medical Center, Ramat Gan, Israel; Obstetrics & Gynecology Department, IVF Institute, Sheba Medical Center, Ramat Gan, Israel; Faculty of Medical and Health Science, Tel Aviv University, Tel Aviv, Israel; Obstetrics & Gynecology Department, Fertility Preservation Center, Sheba Medical Center, Ramat Gan, Israel; Obstetrics & Gynecology Department, IVF Institute, Sheba Medical Center, Ramat Gan, Israel; Faculty of Medical and Health Science, Tel Aviv University, Tel Aviv, Israel

**Keywords:** fertility preservation, cancer survivors, childhood cancer survivors, childhood cancer, IVF

## Abstract

**STUDY QUESTION:**

What are the outcomes of controlled ovarian stimulation (COS) in childhood cancer survivors (CCS) undergoing fertility preservation (FP) after cancer treatment?

**SUMMARY ANSWER:**

CCS who have undergone chemotherapy often show poor outcomes with COS and may need multiple cycles to achieve an adequate number of oocytes for future pregnancy.

**WHAT IS KNOWN ALREADY:**

Up to 65% of CCS experience infertility from gonadotoxic treatments. Although it is ideal to consider FP at diagnosis, age and oncological factors often limit this option. After recovery, pubescent survivors, especially those who could not preserve fertility earlier, may be offered oocyte cryopreservation.

**STUDY DESIGN, SIZE, DURATION:**

A retrospective study including 20 CCS who underwent COS for oocyte storage between 2015 and 2022.

**PARTICIPANTS/MATERIALS, SETTING, METHODS:**

This study involved young CCS who had been previously treated with chemotherapy and were evaluated at an FP center in a tertiary medical center. CCS were encouraged to pursue endocrine surveillance after recovering from cancer and were offered oocyte storage in case diminished ovarian reserve was evident, as dictated by elevated basal FSH (>10 IU/l), decreased anti-Müllerian hormone (AMH; <25th percentile for age), or low antral follicle count (<7).

**MAIN RESULTS AND THE ROLE OF CHANCE:**

Mean age at cancer diagnosis was 13.24 ± 5.6 years. Seventeen patients (85%) had been treated with alkylating agents, with five receiving cumulative doses greater than 4000 mg/m^2^. At the time of FP, a median of 4.25 years after cancer diagnosis, the mean age of patients was 20.6 ± 3.56 years. Mean Day 3 FSH levels were 9.26 ± 3.4 IU/l, and 12 patients had AMH levels below 1 ng/ml. The first stimulation cycle lasted 9.4 ± 2.1 days, with a mean gonadotropin dose of 3246 ± 1057 IU and a median peak estradiol (E2) level of 3733 pmol/ml (IQR 1424–6796). The median number of oocytes retrieved in the first stimulation cycle was 5.5, with a median of four mature oocytes. By the end of the FP process, which involved 1–7 cycles per patient, the median number of oocytes stored was 13.5 (IQR 3.5–18.5). Twelve patients managed to store more than 10 oocytes.

**LIMITATIONS, REASONS FOR CAUTION:**

The study is exploratory in its nature, limited by its small sample size and its retrospective design.

**WIDER IMPLICATIONS OF THE FINDINGS:**

Oocyte storage is feasible yet limited in young CCS. Despite their young age at the time of FP, CCS who have undergone chemotherapy often show poor outcomes with COS. Ongoing reproductive monitoring after recovery is crucial to identify those who would benefit from FP following cancer treatment.

**STUDY FUNDING/COMPETING INTEREST(S):**

The Fertility Preservation Unit funds (Sheba Medical Center) were used to support the authors throughout the study period and manuscript preparation. None of the authors declare any conflicts of interest.

**TRIAL REGISTRATION NUMBER:**

N/A.

WHAT DOES THIS MEAN FOR PATIENTS? Previous studies have shown that up to 65% of childhood cancer survivors, who had undergone treatments affecting egg or sperm production, experience infertility. Storage of oocytes (eggs) at the time of cancer diagnosis is often not possible for girls due to their age and characteristics of the disease; however, it can be offered after recovery, giving the patients a chance to have biologic offspring in the future.

This study looked back at outcomes of controlled ovarian stimulation, a method of promoting oocyte development to collect and storing the eggs for future use, after recovery from a childhood cancer. This study showed that most patients (12/20) managed to store a total of more than 10 oocytes. Patients who have recovered from childhood cancer should be informed about the option of post-cancer fertility preservation through oocyte storage. Monitoring of ovarian function should begin in adolescence to detect possible diminished ovarian reserve, which may then prompt consideration of oocyte storage as a potential option.

## Introduction

Each year, around 400 000 individuals under the age of 20 receive a cancer diagnosis (Steliarova-Foucher *et al.*, 2017). While more than 80% of these young patients can now be cured (Lam *et al.*, 2019), many will confront long-term effects of treatment, with infertility emerging as a prominent concern (Nicholson and Byrne, 1993; Sklar *et al.*, 2006). Infertility rates among cancer survivors vary greatly depending on the type of cancer treatment administered. Generally, up to 65% of childhood cancer survivors (CCS) may experience infertility (Lehmann *et al.*, 2019), but this rate increases to 80% for those who receive hematopoietic stem cell transplantation (HSCT) (Borgmann-Staudt *et al.*, 2012).

Maintaining the potential to have biological children in the future is closely tied to improving quality of life. The desire to have a child is estimated to be present among 90% of pubescent cancer patients who had been treated for cancer (El Alaoui-Lasmaili *et al.*, 2023), emphasizing the critical importance of fertility preservation (FP). When considering FP in the pediatric population, specific challenges arise. The emotional burden of fertility-related procedures at such a young age can be overwhelming for both the patients and their parents (Baysal *et al.*, 2015). Many fear treatment delay (Kohler *et al.*, 2011) or prefer to focus on cancer treatment (Wyns *et al.*, 2015), potentially leading them to decide against pursuing FP altogether. For those who do opt for FP, ovarian stimulation and oocyte storage are often impractical due to critical medical condition, oncologic time constraints (Benedict *et al.*, 2015), or a patient’s prepubescent status. In such scenarios, ovarian tissue cryopreservation (OTC) remains an option (Shapira *et al.*, 2020; Dolmans *et al.*, 2021), although there is concern that future transplantation could be hindered by potential contamination of the tissue with cancer cells (Dolmans *et al.*, 2016; Shapira *et al.*, 2018).

It is crucial to note that, in certain instances, the opportunity for FP extends beyond the timing of cancer diagnosis. After recovering from cancer, some patients may experience primary ovarian insufficiency (POI), while others maintain normal ovarian function. In between these categories, there are patients with diminished ovarian reserve (DOR) who could potentially benefit from oocyte storage after puberty (Filippi *et al.*, 2021), though data regarding this option, for this specific population, are sparse. In this study, we aimed to evaluate the outcome of ovarian stimulation performed in CCS for the purpose of oocyte cryopreservation.

## Materials and methods

### Study population

This study involved 20 CCS treated at the FP unit of a tertiary medical center (The Sheba Medical Center, Ramat-Gan, Israel). Our clinical database was searched for cancer survivors undergoing ovarian stimulation for oocyte storage between 2015 and 2022. Inclusion criteria were previous exposure to chemotherapy and age younger than 21 years at the time of cancer diagnosis. Exclusion criteria were urgent oocyte storage in the face of disease relapse or secondary malignancy and pelvic radiation.

### Patient management

The FP center serves newly diagnosed prepubertal and post-pubertal cancer patients, as well as cancer survivors seeking post-cancer fertility care. Medical awareness of FP after cancer treatment has only started to gain notice in recent years. Since 2015, we have been advising cancer patients who are post-pubertal and over the age of 14 to schedule a post-cancer consultation at the FP center, 1 year after completing cancer treatment. Younger patients are typically monitored and cared for by the pediatric endocrinology team. Referral to our clinic takes place starting 2 years after menarche. Girls experiencing hypogonadotropic hypogonadism along with DOR are referred to our clinic around the age of 14. At post-cancer FP consultation, ovarian reserve tests are evaluated, and oocyte storage is considered accordingly. Oocyte storage is suggested in case DOR is evident, as dictated by elevated basal FSH (>10 IU/l), decreased anti-Müllerian hormone (AMH; <25th percentile for age), or low antral follicle count (AFC) (<7). Patients are advised against FP in cases where FSH levels exceed 20 IU/l. Considering the cohort’s young age, the 25th percentile aligns with an AMH serum level of ∼2.6 ng/ml. AFC is usually not assessed in subjects who did not have prior sexual intercourse. Patients receiving combined estrogen–progestin therapy are instructed to discontinue hormonal treatment before undergoing evaluation of ovarian reserve.

### Ovarian stimulation protocol

The multiple-dose GnRH antagonist protocol was used in all patients for their first stimulation cycle. The modified natural cycle–IVF protocol was used in repeat cycles, for patients who had previously shown poor response of 1–2 oocytes per cycle. The selection of gonadotrophin type was made by the treating physician. Gonadotrophin doses were administrated in variable doses (with a minimal daily dose of 300 IU), and further adjusted based on ultrasound scan and serum estradiol (E2) levels obtained every 2–3 days. Final follicular maturation was induced when the leading follicle measured >17 mm, with either hCG (250 mcg Ovitrelle, Merck), GnRH-agonist (GnRH-a) (Decapeptyl 0.2 mg), or dual trigger (Ovitrelle 250 mcg and Decapeptyl 0.2 mg). In general, as all patients were designated for oocyte storage, GnRH-a was the default triggering agent. Triggering with r-HCG or dual trigger (rHCG+GnRHa) was considered in the presence of prolonged oral contraceptives usage and possible hypothalamic–pituitary axis dysfunction (secondary to previous CNS radiation). A transvaginal, ultrasound-guided follicular aspiration was conducted 36 h after triggering. The number of cycle attempts for each patient was determined through a shared decision-making approach. After each ovum pick-up, a follow-up meeting was held to discuss the outcomes of the previous cycle and to present potential next steps. The goal was typically to preserve a minimum of 10 oocytes in total, taking into consideration the patient’s individual ovarian response, and personal family planning preferences. Financial factors frequently influenced the decision-making process; whenever two reduced ovarian reserve indices had been present, the national public health system covered up to four cycles or until 20 oocytes had been stored, whichever came first.

### Data collection

Institutional ethics committee approval was obtained for the study. Electronic medical records were reviewed to extract demographic, oncologic, and reproductive data. Oncologic data included cancer diagnosis, chemotherapy protocol, utilization of radiotherapy, surgical intervention, and application of hematopoietic stem cell transplantation (HSCT). To evaluate the extent of the treatment gonadotoxicity, the cumulative doses of alkylating agents administrated were recorded into the cyclophosphamide equivalent dose (CED) calculator (Green *et al.*, 2014b). A CED value greater than 4000 mg/m^2^ was considered to represent a notable risk of infertility (Green *et al.*, 2014b). Reproductive data included previous FP procedures upon diagnosis, menstrual pattern at the time of post-cancer FP, ovarian reserve indices, and ovarian stimulation characteristics and outcomes. Stimulation outcomes (number of retrieved and mature M2 oocytes) served as the primary outcome measure. ‘Actual time lapse’ between cancer diagnosis and timing of post-cancer FP was calculated by subtracting the date of the former from the latter. As 14 years of age represents the earliest age at which ovarian stimulation and ovum pick are performed in our center, a ‘Relevant time lapse’ was defined. For patients older than 14 years at cancer diagnosis ‘relevant time lapse’ was identical to ‘actual time lapse’, whereas for patients younger than 14 years at diagnosis, the relevant time lapse was calculated subtracting 14 from the age at post-cancer FP.

### Statistics

Statistical analysis was conducted with the use of SciPy, version 1.0.0 (Virtanen *et al.*, 2020). Normality of distribution was evaluated with the use of histograms and Q–Q plots. Continuous variables are presented as mean ± SD or median (IQR) as appropriate. Categorical variables are presented as n (%).

## Results

Between 2015 and 2022, 57 cancer survivors who had been previously treated with chemotherapy underwent ovarian stimulation for oocyte storage. Thirty-six of them were over the age of 21 at the time of cancer diagnosis, and one presented for FP at the time of secondary cancer diagnosis. Thus, a total of 20 patients were included in the study. Their characteristics are presented in Table 1. Six patients recovered from leukemia, nine from lymphoma, three from CNS tumor, one from germ cell tumor, and one from neuroblastoma. Mean age at cancer diagnosis was 13.24 ± 5.6 years. Six patients were pre-menarcheal at diagnosis, and 14 were post-menarcheal. Nine patients underwent FP using OTC around the time of cancer diagnosis. Seventeen patients (85%) received alkylating agents, five of whom were treated with CED >4000 mg/m^2^, seven were treated with a CED lower than 4000 mg/m^2^, four were treated with Dacarbazine (3000–7000 mg/m^2^), and one with Treosulfan (54 000 mg/m^2^). The two latter treatments are both alkylating agents not included in the CED calculation formula. Seven patients received HSCT, and four were treated with brain irradiation.

**Table 1.** Patients’ characteristics on diagnosis and post-cancer FP.

	Cancer diagnosis and treatment								Post-cancer FP				
	Age (years)	Diagnosis	FP at diagnosis	CED (mg/m^2^)	Other gonadotoxic chemotherapies	AA	HSCT	Rx	Age (years)	Menses	AFC	D3FSH (IU/l)	AMH (ng/ml)
**Poor response (1st cycle)**													
1	15	HL	OTC	3700	Dacarbazine (1500)	Y	Y		27	Irregular	4	14.3	0.03
2	20	HL		0	Dacarbazine (3000)	Y	N		23	OCP		4.6	0.54
3	9.5	Optic glyoma		0	Carboplatin (7000)	N	N	CNS	25	Amenorrhea		7.6	1.4
4	13	Meduloblastoma	OTC	12 000	Cisplatin (375)	Y	N	CNS	20	Irregular		8.8	0
5	20	NHL	OTC	2250		Y	N		21	Regular		12.7	0.48
6	16	Meduloblastoma	OTC	13 200	Cisplatin (450)	Y	Y	CNS	21	Regular		16	0.06
7	1.8	Neuroblastoma		15 800		Y	Y		15	Irregular		13.5	0.02
8	2	ALL	OTC	NA	NA	NA	Y		15	Regular		6.97	0.13
9	13	ALL	OTC	8700		Y	Y	TBI	25	Regular	4	14.5	0.46
**Suboptimal response (1st cycle)**													
10	19	HL		0	Dacarbazine (4500)	Y	N		21	Regular	18	8.5	8.6
11	18	HL		0	Dacarbazine (3000)	Y	N		21	Regular	3	9.7	0.16
12	6	ALL		3000		Y	N		27	Regular		10.4	1.98
13	14.5	AML	OTC	0	Treosulfan (52 000), fludarabine (600)	Y	Y		19	OCP		7.6	1.04
**Normal response (1st cycle)**													
14	19	HL	OTC	0	Dacarbazine (4500)	Y	Y		20	Regular	7	12	0.94
15	17	HL		1000	Dacarbazine (750)	Y	N		19	Regular		6.2	1
16	16	NHL		3150		Y	N		18	Regular		6.3	4.96
17	12	Ovarian germ cell tumor		0	Cisplatin (400)	N	N		16	Irregular	10	8	0.92
18	4	ALL		3000		Y	N		24	OCP		4.9	1.42
19	15	ALL		5500		Y	N	CNS	18	Regular		4.89	1.42
20	14	HL	OTC	2000	Dacarbazine (1500)	Y	N		17	Regular		7.7	1.2

HL, Hodgkin lymphoma; NHL, non-Hodgkin lymphoma; ALL, acute lymphocytic leukemia; AML, acute myeloid leukemia; FP, fertility preservation; OTC, ovarian tissue cryopreservation; CED, cyclophosphamide equivalent dose; AA, alkylating agents; HSCT, hematopoietic stem cell transplantation; Rx, radiation; CNS, central nervous system radiation; TBI, total body irradiation; OCP, oral contraceptives; AFC, antral follicle count; D3FSH, Day 3 FSH; AMH, anti-Müllerian hormone; Y, yes; N, no; NA, data not available.

Mean age at FP after recovery from cancer was 20.6 ± 3.56 (range 15–27). Median ‘Actual time lapse’ between cancer diagnosis and timing of post-cancer FP was 4.25 years (range 1–21), whereas median ‘relevant time lapse’ was 3 years (range 1–12 years). At the time of FP, 12 patients had regular menstruation, four had oligomenorrhea, three were taking oral contraceptives, and one had amenorrhea.

All patients except for two showed DOR according to at least one sonographic/laboratory marker. Patients #10 and #16 did not show any signs of DOR. Nonetheless, both expressed a very strong will for FP, mostly due to the fear of disease relapse which would require additional, more intense, chemotherapy treatment. Mean FSH levels were 9.26 ± 3.4 IU/l. Seventeen patients had AMH levels lower than 2.6 ng/ml (<25th percentile for age) and 12 patients had AMH levels lower than 1 ng/ml.

**Figure 1.**
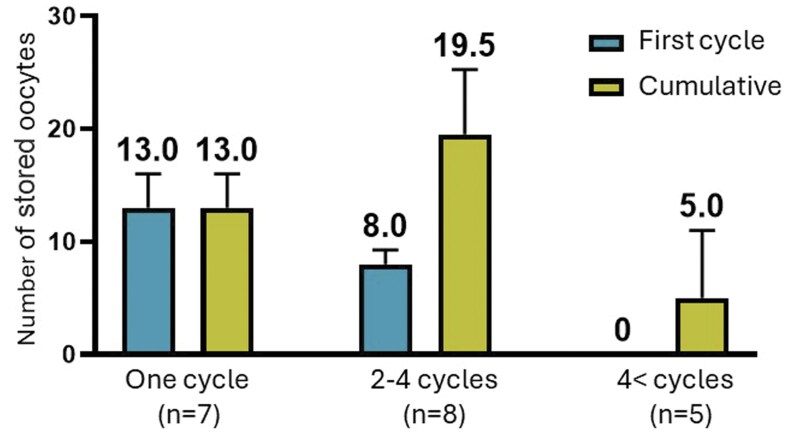
Number of stored oocytes, according to total number of cycles performed per patient.

The first stimulation cycle attempt lasted an average of 9.4 ± 2.1 days with a mean cumulative gonadotropins dose of 2977 ± 1019 IU. Median maximal E2 levels reached 3733 pmol/ml (IQR 1424–6796). GnRH-a was used for triggering in 75% of patients, HCG trigger in 15% (patients #2, #3, #18), and dual trigger in 10% (patients #4, #19). The median number of oocytes retrieved on the first stimulation cycle was 5.5, and the median number of mature oocytes was four. Nine patients demonstrated poor ovarian response (<4 retrieved oocytes) during their first cycle, and additional four demonstrated a sub-optimal ovarian response (4–9 retrieved oocytes). Of these 13 patients, four had been exposed to CED of over 4000 mg/m^2^, while the remaining nine patients had been exposed to lower CED, or to chemotherapy protocols deprived of agents included in the CED calculation formula. Six of these patients had received HSCT. Seven of the poor/suboptimal responders underwent OTC on cancer diagnosis. Additional data according to ovarian response in the first cycle are presented in Table 1. By the conclusion of the FP process, the median number of stored oocytes was 13.5 (IQR 3.5–18.5). Twelve patients had stored more than 10 oocytes. As shown in Fig. 1, seven patients chose to undergo a single stimulation cycle, eight patients opted for 2–4 stimulation cycles, and five patients elected to undergo more than four stimulation cycles each. The median cumulative number of stored oocytes at the end of FP process was 13, 19.5, and 5, respectively, for these groups.

## Discussion

In this study, FP for CCS after completion of cancer treatment resulted in a median of four cryopreserved oocytes during the initial stimulation cycle. Through repeated cycles, participants accumulated a median of 13.5 oocytes by the conclusion of the FP process. More than half of the study’s cohort managed to cryopreserve over 10 oocytes for future use, underscoring the feasibility of post-childhood cancer FP.

Our data highlight the potential value of post-cancer endocrine/fertility surveillance among CCS. At the time of FP, only 5 of 20 patients experienced menstrual irregularities, possibly prompting them to seek medical advice. Conversely, for the remaining patients, decline in ovarian reserve and chance for FP could go unnoticed without a dedicated medical/endocrine monitoring service. The significance of such monitoring becomes more apparent when we consider the rate of poor or suboptimal response observed in the study group (65%), especially given the young age of the patients, who may wish to defer conception for a considerable period. Though current literature lacks data concerning outcomes of ovarian stimulation in CCS, several studies investigated the proportion of CCS who exhibit DOR and may benefit from oocyte cryopreservation. In one study, rates of DOR and POI among 80 young cancer survivors were 26% and 64%, respectively (Lehmann *et al.*, 2020). Eleven subjects with DOR (52%) stored their oocytes. In a more recent study, out of 126 CCS undergoing post-cancer reproductive surveillance, 39% were diagnosed with DOR and 21% with POI (Filippi *et al.*, 2021). Altogether, the high incidence of DOR among CCS, along with the demonstrated feasibility of oocyte storage in the current study, underscores the importance of an ongoing reproductive monitoring following cancer treatment.

Previous cancer treatments may indicate which patients could benefit the most from such monitoring. A notable decrease in ovarian reserve is more probable after undergoing HSCT and high-dose alkylating agent therapy. The latter is often quantified by CED, where a CED greater than 4000 mg/m^2^ is linked to a higher risk of premature menopause (Green *et al.*, 2014b). Yet, in our study, out of the 13 patients who showed poor or suboptimal response, nine had either not been exposed to alkylating agents, had received lower cumulative equivalent alkylating agent doses (CED < 4000 mg/m^2^), or had undergone chemotherapy protocols that did not include alkylating agents factored into the CED formula (e.g. dacarbazine, a non-classical alkylating agent). Indeed, it has been demonstrated that despite the standardization of alkylating agent dose, a significant degree of interpatient variability still exists. The St Jude Lifetime Cohort Study illustrated that while the risk of gonadotoxicity rises with higher cumulative doses, there is no minimum threshold below which individuals are considered safe from the risk of infertility (Green *et al.*, 2014a). Despite being at negligible risk for ovarian hyperstimulation syndrome, most patients in the current cohort received a GnRH-a trigger. The decision to use the GnRH-a trigger was based on the potential benefits of endogenous LH and FSH production resulting from the GnRH-a trigger. Previous studies have shown that GnRH-a is associated with a higher rate of mature oocytes compared to hCG (Humaidan *et al.*, 2012; Herzberger *et al.*, 2021), likely due to this physiological response. In selected cases, such as patients who were more prone to suboptimal GnRH agonist response (prolonged OCP use, previous CNS radiation) (Meyer *et al.*, 2015), HCG or dual trigger were administrated.

In our study, nearly half of the patients opted for post-cancer oocyte storage even though they had already stored ovarian tissue at the time of cancer diagnosis. The success rates of OTC and transplantation are now well-established, with estimates suggesting up to a 40% chance of achieving at least one live birth (Shapira *et al.*, 2020). However, it remains advisable to offer additional FP options after recovery, particularly for CCS. Many of these patients recover from malignancies that are more commonly linked to ovarian tissue contamination with cancer cells (Shapira *et al.*, 2018; Grubliauskaite *et al*., 2023), which contraindicates auto-transplantation. Additionally, evidence for the successful transplantation of tissue obtained before puberty is still limited (Demeestere *et al.*, 2015; Matthews *et al.*, 2018). Therefore, we advocate for post-cancer fertility monitoring, regardless of the type of previous oncologic treatments or prior OTC. Of note, seven out of the nine patients who had received OTC responded poorly/sub-optimally. This figure cannot be interpreted as evidence of a causal relationship, as those who received OTC were more likely to have been treated with more gonadotoxic therapies to begin with. For instance, five of these patients had received bone marrow transplantation. Most OTC recipients in the study underwent partial oophorectomy. However, even when the entire ovary is removed, the impact on ovarian reserve is not straightforward. Logically, removing 50% of the ovarian reserve should result in the depletion of the follicle pool much earlier than in women with two ovaries. However, this does not seem to be the case. Several studies have shown that menopause occurs only 1–1.5 years earlier following unilateral oophorectomy (Rosendahl *et al.*, 2017; Gasparri *et al.*, 2021). A recent meta-analysis comparing infertile women who underwent unilateral oophorectomy with women who have two intact ovaries undergoing IVF found that unilateral oophorectomy negatively impacted oocyte yield, with a weighted mean difference of only −2 aspirated oocytes per cycle (Younis *et al.*, 2018). Interestingly, in patients with two ovaries, both AFC and oocyte yield per ovary were significantly lower than those observed in patients who had undergone unilateral oophorectomy, indicating a possible compensatory mechanism (Khan *et al.*, 2014). With regard to our study, it is not possible to isolate the effect of OTC (mainly partial oophorectomy) on ovarian response. It should be taken into consideration that the use of standard markers of ovarian reserve to predict response to ovarian stimulation in adolescents is still unclear (Anderson *et al.*, 2022), and discrepancies between unfavorable test results and the number of cryopreserved oocytes have been reported in the past (Slonim *et al.*, 2023). In the current study, AMH levels were below 1 ng/ml in 12 out of 20 patients. We could not evaluate the association between AMH levels and ovarian response due to the limited size of our cohort.

Despite the young age of the current cohort, due to poor/suboptimal ovarian response, most patients underwent multiple cycles in pursuit of a favorable chance for future pregnancy. At the extremes, five patients had undergone more than four cycles each. As shown in Fig. 1, none of them were able to store even a single oocyte after their first cycle. Consulting a very young patient whose first cycle resulted in no mature oocytes is, at the very least, emotionally challenging. Their poor prognosis should be communicated clearly, yet gently, and the option of future pregnancy with an oocyte donor should be presented as a viable alternative. However, these patients often understand that POI is impending and, in our experience, are frequently determined to undergo another cycle, which typically leads to multiple additional attempts. At this stage, the antagonist protocol is often switched to a modified natural protocol, which not only reduces costs but also improves treatment tolerability and safety.

One could argue that initiating treatment earlier could enhance the yield of oocytes per cycle. We observed that the median ‘relevant time lapse’ in the current cohort was 3 years. This figure is reasonable, considering that certain oncological treatments extend over several months, and post-cancer FP typically begins no sooner than 6 months after chemotherapy (Chung *et al.*, 2013). Nonetheless, a wide range of time lapses has been noted in this cohort (1–12.5), and for at least some patients, earlier intervention might have potentially overcome further time-related decline in ovarian reserve. Such potential advantage should be weighed against the limited data regarding pregnancy outcomes from oocytes stored for very young patients. We found only one study documenting a pregnancy and live birth following long-term cryopreservation of oocytes, involving a 17-year-old patient undergoing gonadotoxic treatment for pulmonary hypertension (Kim and Hong, 2011). Additionally, higher rates of fetal aneuploidy have been described in adolescent pregnancies, when compared with women in their twenties (Franasiak *et al.*, 2014).

The idea of oocyte cryopreservation during the early stages of the reproductive lifespan is further challenged by studies assessing the competency of oocytes collected from young girls. A pivotal study on small antral follicles from ovarian tissue of unstimulated girls and women before chemotherapy has demonstrated that meiosis I nondisjunction events are linked to a higher incidence of aneuploidy in oocytes from young girl (Gruhn *et al.*, 2019). Another study has shown that a maturational process occurs within the ovary during transition from childhood through puberty and adulthood. This process results in the loss of large number of abnormal follicles and oocytes which are found to a much higher extent in the young ovary (Anderson *et al.*, 2014).

The emotional toll of performing ovarian stimulation at a young age should also be taken into consideration. When dealing with young patients, especially adolescents, FP introduces various ethical issues, including concerns about consent and cultural or religious implications. For example, the possibility of unintentional hymenectomy during oocyte retrieval can be particularly distressing for virginal patients (Sönmezer *et al.*, 2023). These considerations need to be approached with great sensitivity when discussing FP options. Furthermore, the demands of frequent monitoring and multiple daily injections can be difficult for a young adolescent to manage. Fertility treatment procedures are generally low risk, but adolescents may still endure significant distress, discomfort, or pain during their treatment.

Our study expands our knowledge, demonstrating the feasibility of oocyte storage for CCS, though few limitations should be acknowledged. First, due to the small sample size, statistical analysis could not be performed to evaluate the association between various clinical parameters and oocyte yield. Given the increasing awareness of post-cancer fertility care, we expect that larger studies will allow for the evaluation of these associations in the future. Second, because a single marker of compromised ovarian reserve was deemed sufficient to recommend oocyte storage, and evaluating AFC abdominally was challenging for some virginal patients, many patients were not assessed for AFC. As a result, this information was missing for most of the included patients. Finally, we were unable to provide data on pregnancies and live births, which are of particular interest considering the young age of some patients included in this study.

To conclude, this study underscores the rationale for post-cancer reproductive surveillance. Our data indicate that oocyte storage is a viable option for CCS, but it also highlights significant variability in ovarian response among these young patients. Consequently, the number of cycles required to secure a favorable chance of future pregnancy can differ substantially from one patient to another. Until further data become available, we recommend post-cancer reproductive surveillance for all young cancer survivors, irrespective of previous oncologic therapies. Oocyte storage should be considered according to patient’s age, ovarian reserve status, emotional maturity, and family planning considerations.

## Data Availability

The data underlying this article will be shared upon reasonable request to the corresponding author.
